# The pain medicine fellowship landscape, an observational analysis

**DOI:** 10.1016/j.inpm.2022.100086

**Published:** 2022-03-25

**Authors:** Taif J. Mukhdomi, Daniel R. Vanzant, Marcus D. Harris, Junaid J. Mukhdomi, Benjamin D. Mirman, Parker A. Woolley, Mark C. Kendall

**Affiliations:** a Department of Pain Medicine, Memorial Sloan Kettering Cancer Center, New York, NY, United States; b Department of Pain Medicine, Weill Cornell College of Medicine, New York, NY, United States; c Department of Anesthesiology, Cleveland Clinic, Cleveland, OH, United States; d Department of Anesthesiology and Pain Medicine, University of Michigan, Ann Arbor, MI, United States; e Department of Anesthesiology and Pain Medicine, Rush University, Chicago, IL, United States; f The Warren Alpert Medical School, Brown University, Providence, RI, United States; g Department of Anesthesiology, Brown University, Providence, RI, United States

## 1 Introduction

In an era when the prevalence of chronic pain is predicted to rapidly expand, the need for pain medicine physicians is apparent more than ever. The demand for pain physicians has been recently highlighted in Congress with the implementation of the Opioid Workforce Act in 2019, advocating for more pain medicine fellowship positions, with an aggregate increase of 1,000 positions over the next five years. The pain medicine fellowship match process continues to be competitive as it is one of the only subspecialty fellowships that is multidisciplinary, which diversifies the selection process. The fellowship positions are pursued not only by anesthesiology residents but also physiatry, neurology, psychiatry, and other qualified residents, thus complicating the match process and increasing competitiveness to achieve postgraduate pain medicine training. The need for pain providers is expected to continue to rise and far outpace the training of new pain medicine providers, however, despite the anticipated increase in pain providers in the next half decade. Pain care delivery has struggled with increasing demand compounded by insufficient access to care [1].

Even with the growing need for pain physicians and the expected increase in fellowship positions, limited studies have been performed to identify notable trends in the pain medicine fellowship match. This includes various factors that may influence obtaining an interview and matching at a program. One such factor is the number of yearly applicants compared with the number of positions offered; it is vital to have an understanding how current and future interest in the field compares with the relative positions available, among other patterns in the application and match process, in order to optimize the pain medicine fellowship training process and better shape the future of the field.

The National Resident Matching Program (NRMP) surveyed pain medicine program directors in 2016 and found that the top characteristics of applicants are: perceived interest in specialty/program, letters of recommendation, USMLE/COMLEX Step 1 scores and demonstrated interest in research [2]. Requirements of fellowship applications include residency performance, letters of recommendation, medical school performance, Medical Student Performance Evaluation (MSPE), USMLE scores, qualities of leadership, and scholarly achievements as metrics for evaluating fellowship applicants. Interestingly, no role of geographic location was noted [3,4]. There have been several investigations exploring the importance of geographic location in the match process to other specialties [[5], [6], [7], [8], [9], [10]]. One study demonstrated that a significant proportion of applicants (24.6%) matched at a program affiliated with their medical school. This phenomenon was found to be significant especially in states with 2 or fewer medical schools [5]. Another recent study in orthopedic surgery residency also revealed geographic bias within the match process, with a significant proportion of residents (21%) remaining in the same region as their medical schools. More pertinently, residency program location may play a significant role in the fellowship match for pain medicine applicants. More than half (54.6%) of the individuals who completed residency training from 2009 through 2018 are practicing in the state where they did their residency training [11]. Since these regional trends occur for where residents go on to practice, it is plausible the same holds true for where residents go on to train as fellows. To our knowledge, however, there have been no investigations focused on the role of geographic location specifically in the pain medicine fellowship application and matriculation process. Considering these findings and the relative paucity of data relevant to pain medicine applicants, one goal of our present analysis is to evaluate geographic trends among matriculated pain medicine fellows.

Our objective in this study was to bring to light important trends in the pain medicine fellowship match process that could help future programs and applicants. This includes the association between the location of applicant residency training program and the location of matched fellowship applicant program. In addition, we also examined the relationship between medical school location and its relation to matched fellowship location, gender, medical degree type, and the amount of fellowship positions offered compared with the corresponding applicant pool size over the past seven years.

## 2 Methods

For the portion of the study comparing fellowship positions offered and applicant pool size, data on the pain medicine fellowship match of residents was extracted from the National Resident Matching Program (NRMP), Results and Data: Specialties Matching Service between 2014 and 2020 [2]. The Strengthening the Reporting of Observational Studies in Epidemiology (STROBE) guidelines were used to ensure the reporting of this observational study. Data on residents were extracted based on medical degree, successful match, and unmatched status. Data on programs were also extracted based on applicant type and unfilled programs. This comparison was performed for each academic year and for all years combined (2014–2020). Data are presented as counts and percentages and analyzed using the chi-square test of independence. Statistical significance was accepted at P ​< ​0.05. All statistical analysis was performed using Stata ver.11 (Stata Corp., College Station, TX).

Our study also reviewed interviewee and residency program data after being gathered from department websites and 4 software platforms (Doximity, LinkedIn, ACGME, and US News and World Report) and de-identified before analysis. The Fellowship and Residency Electronic Interactive Databases (FREIDA), (http://www.ama-assn.org/ama/pub/education-careers/graduate-medical-education/freida-online.page), was used to identify current pain medicine fellowship programs. The departmental websites were then accessed through the FREIDA online system or, if not available, through an Internet search engine. This study qualified as nonhuman subject research and was thus exempted from institutional review board (IRB# 1593835).

From this database, 104 pain medicine fellowship programs were identified, 97 of which were included in the study. The inclusion criteria were (1) availability of a roster of the current pain medicine fellows and (2) a listing of what medical schools the residents attended. Of the 7 programs excluded, 1 program had closed, while another was just recently accredited (n=1) or were missing any data of the current fellows (n=5). In this study, research fellowships and other fellowship positions were not included in the data.

If a program had an incomplete listing of the fellow’s training history, a Google, Doximity, and LinkedIn search was performed to identify the fellow’s education background. If this information was not available, the remainder of the program’s information was not utilized in the study. Furthermore, residency programs with no affiliated medical school were not included in the study. The medical school affiliation for each program was obtained through the FREIDA online database or through departmental websites. The number of medical schools within the state for each program was determined through the Association of American Medical Colleges Electronic Residency Application Service website (https://www.aamc.org/services/eras/).

For the 97 programs included, the region of the program location was identified through the FREIDA online database (New England, Mid Atlantic, East North Central, West North Central, South Atlantic, East South Central, West South Central, Mountain, and Pacific). The same categories were applied to the associated medical schools. This led to the following data being collected: (1) characteristics of fellowship programs, (2) regions of fellowship programs, (3) characteristics of current fellows, (4) numbers of fellows from the affiliated residency/medical school programs, and (5) numbers of fellows from schools in all geographical regions.

The programs to which these trainees matched were determined from the ACGME-accredited programs from the following publicly available resources: program trainee lists, published medical school match lists, interviewees’ Doximity profiles, LinkedIn profiles, and/or Google searches of name with terms such as “pain medicine fellow” or similar. Identifying program features were removed from the dataset.

## 3 Results

In determining geographic patterns in the match, the 2019–2020 academic year of pain medicine fellows showed 37% (119/320) who completed residency at the same institution as they were training for fellowship. Geography has been noted in medical residencies to be an influential factor in the match process. Pain medicine fellows from this same cohort demonstrated similarly notable trends in their demographics and prior training experiences.

### 3.1 Geographical region

There is a clear association between the region where a resident trains and where a resident matriculates into fellowship. Table 1 shows the representation of geographical patterns of retention of pain medicine fellows from their residency training program for the 2019–2020 academic year. The West North Central and Mid-Atlantic regions were the highest with 76% and 75% fellow retention, respectively. The Pacific region was the lowest with a 43% retention.

**Table 1:** The representation of geographical patterns of retention of pain medicine fellows from their residency training program for the 2019–2020 academic year.

Residency training programs	Pain fellowship programs								
	New England	Mid Atlantic	South Atlantic	East North Central	East South Central	West North Central	West South Central	Mountain	Pacific
New England	15/34 (44)	7/34	4/34	3/34	0/34	1/34	3/34	0/34	1/34
Mid Atlantic	3/55	41/55 (75)	*5/55*	4/55	*0/55*	*0/55*	*0/55*	*0/55*	1/55
South Atlantic	2/54	6/54	31/54 (57)	4/54	3/54	1/54	6/54	0/54	1/54
East North Central	1/53	6/53	1/53	37/53 (70)	2/53	3/53	1/53	0/53	2/53
East South Central	0/18	1/18	2/18	1/18	12/18 (67)	0/18	2/18	0/18	0/18
West North Central	0/17	2/17	0/17	1/17	0/17	13/17 (76)	1/17	0/17	0/17
West South Central	0/25	5/25	0/25	1/25	1/25	0/25	16/25 (64)	0/25	2/25
Mountain	0/10	0/10	0/10	3/10	0/10	0/10	1/10	5/10 (50)	1/10
Pacific	1/56	10/56	9/56	6/56	0/56	2/56	2/56	1/56	24/56 (43)

Data presented as number of pain fellows/number of residents. Percent in parentheses. The vertical columns display the pain medicine fellow’s current region of fellowship training. The horizontal columns indicates where these current pain medicine fellows completed their residency training in the United States.

**Fig. 1:**
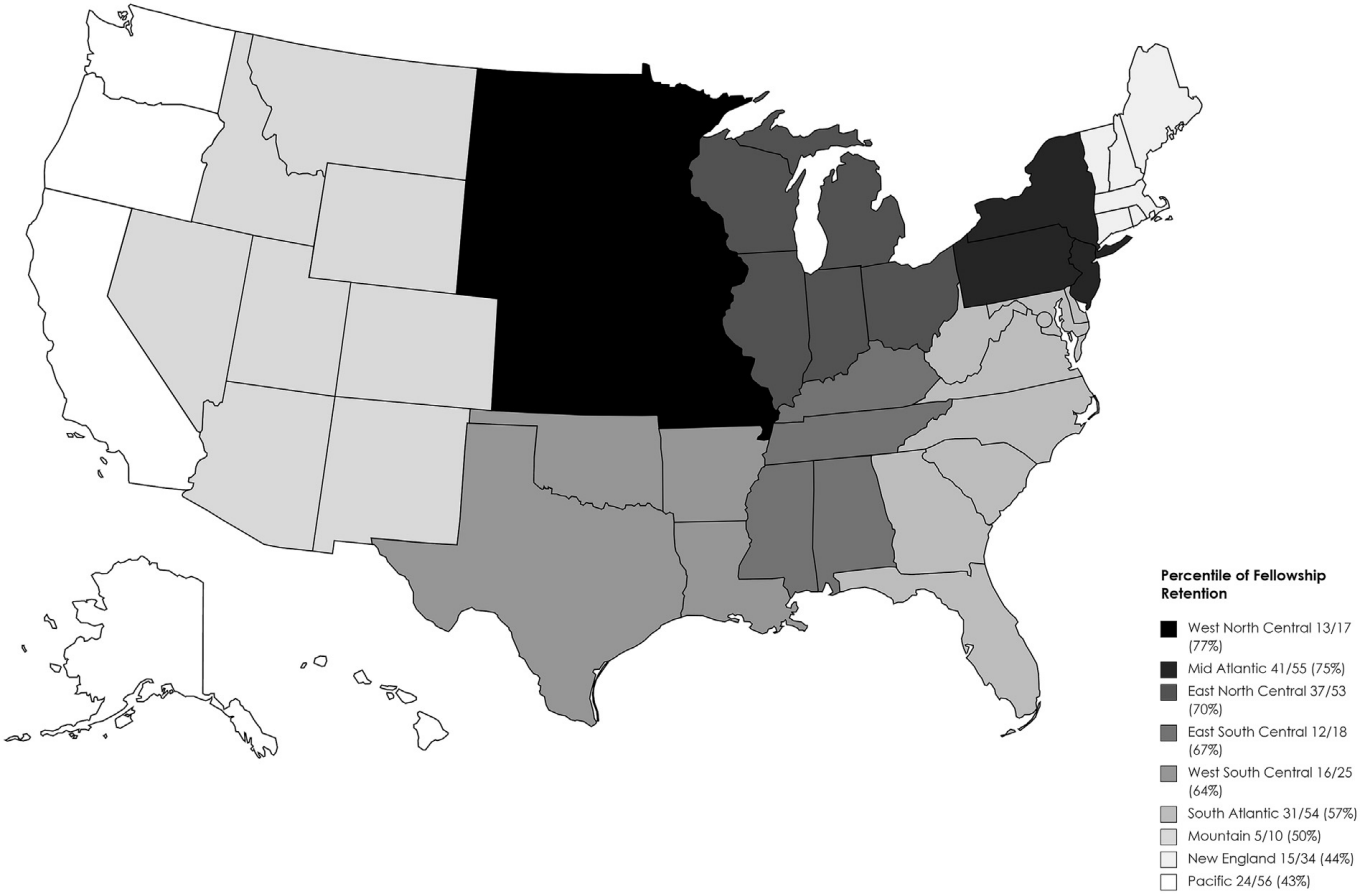
A United States map depicting pain medicine fellow trainee region to residency training region. Legend shows percent of fellows who also completed their residency training within the same region. Data derived from 2019 to 2020 National Resident Matching Program.

A pictorial map of the United States indicating the nine geographical regions where pain medicine fellows completed their fellowship training in the same location as their residency training during the academic year 2019–2020 is displayed in Fig. 1.

### 3.2 State

Data from 2019 to 2020 pain medicine fellowship class, 51% (163/320) completed residency in the same state as they were training for fellowship.

### 3.3 Residency

Data from 2019 to 2020 pain medicine fellowship class, 37% completed residency at the same institution as they were training for fellowship.

### 3.4 Medical school

Data from 2019 to 2020 pain medicine fellowship class, 10.9% were fellows at the same institution as their medical school training.

### 3.5 Demographics

Of the 2019–2020 pain medicine fellows, 77% were male and 23% were female. The pain fellow’s medical degree may also play a role within training programs with 72.5% of pain fellows having a US medical degree (Table 2). When comparing this with the medical degree type for all applicants in this cycle, a total of 55% (285/514) attended US MD-granting medical schools, showing that a higher proportion of US MD applicants are matriculating into pain medicine fellowship.

**Table 2:** Demographic and medical training characteristics of the 2019–2020 pain medicine fellows.

Characteristics	Pain medicine fellows
	(n ​= ​320)
**Gender**	
Male	245 (77)
Female	75 (23)
**Medical Degree**	
United States Graduate	
MD	232 (72.5)
DO	47 (14.7)
Caribbean Medical Graduate	23 (7.2)
Foreign Medical Graduate	18 (5.6)
**Core Medical Specialty**	
Anesthesiology	242 (75.6)
Physical Medicine & Rehabilitation	65 (20.3)
Neurology	6 (1.9)
Psychiatry	1 (0.3)
Other	5 (1.6)

Data presented as n (%). MD ​= ​Doctor of medicine; DO ​= ​Doctor of osteopathic medicine.

### 3.6 Applicant pool size and fellowship positions offered, 2014–2020

In comparing the applicant pool size to the number of NRMP pain medicine fellowship positions offered, our initial search identified a total of 2,915 residents and 2,229 fellowship positions from 2014 to 2020. There were no residents excluded. While the number of positions offered in the match has grown by 40%, the applicant pool has increased by only 8% over the past 7 years (p ​< ​0.014), (Table 3).

**Table 3:** Pain medicine MATCH characteristics for 2014 to 2020 academic years.

	Applicants						
All Applicants, n	398	397	416	401	438	435	430
United States graduates, n	262	266	263	268	287	269	279
Matched, n	256	286	303	309	331	345	361
United States graduates, n	186	196	214	214	231	226	248
Unmatched, n	140	108	112	90	105	90	66
Unmatched, %	35.2	27.2	26.9	22.4	24	20.7	15.3
	**Pain medicine programs**						
NRMP Participants, n	82	84	90	93	98	103	104
Positions offered, n	261	286	305	316	335	359	367
Unfilled positions, n	4	0	2	3	3	8	5
Filled program, %	98.1	100	99.3	97.8	98.8	96.1	98.4
	2014	2015	2016	2017	2018	2019	2020

Data presented as counts or percentages. Data derived from the National Resident Matching Program (NRMP).

### 3.7 Size of program

**Fig. 2:**
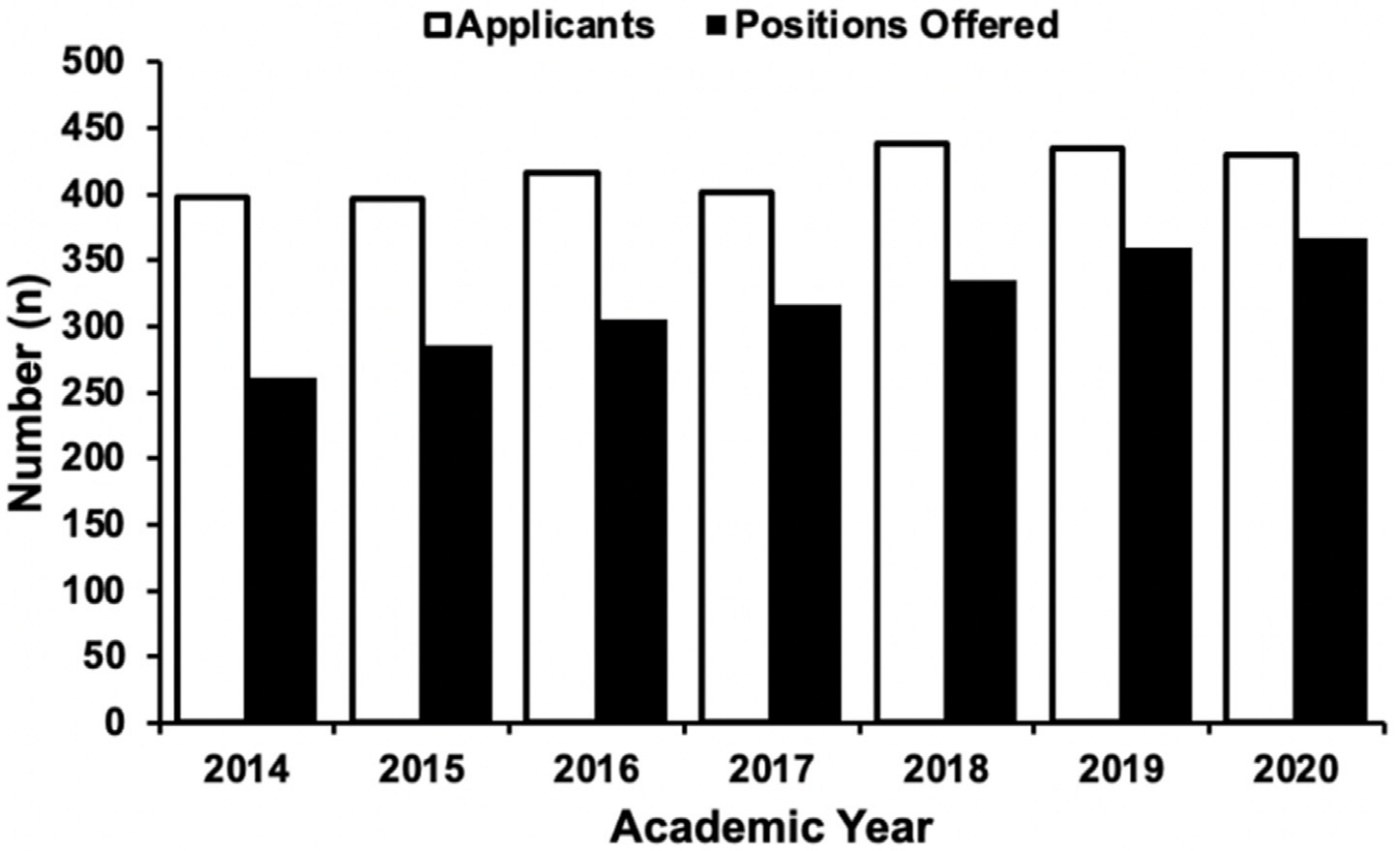
The total of ACGME accredited pain medicine fellowship program applicants and offered positions for the 2014 to 2020.

For the 2019–2020 academic year there are 102 ACGME-accredited pain medicine fellowships. The characteristics of the ACGME-accredited pain medicine fellowships are presented in Table 4. The characteristics of matched and unmatched applicants and positions offered at pain medicine fellowship programs for the academic years 2014–2020 are presented in Table 3. A bar graph indicating the number of pain medicine fellowship applicants and positions offered from 2014 to 2020 is shown in Fig. 2.

**Table 4:** The 2019–2020 ACGME-accredited pain medicine fellowships and respective characteristics.

Characteristics	ACGME -accredited pain medicine fellowship programs in the United States (n ​= ​102)
**Core Department Specialty**	
Anesthesiology	84 (82.3)
Physical Medicine & Rehabilitation	13 (12.7)
Neurology	2 (2)
Pediatric	2 (2)
Closed	1 (1)
**Size of program**	
Small (<= 3 fellows)	41 (40.2)
Medium (4 to 6 fellows)	54 (52.9)
Large (>6 fellows)	7 (6.9)
**Accreditation Year**	
2000 or earlier	65 (63.7)
2001–2010	19 (18.6)
2011-Present	18 (17.7)
**Pain fellowship programs by geographical region**	
New England	9 (8.8)
Mid Atlantic	23 (22.5)
South Atlantic	11 (10.8)
East North Central	19 (18.6)
East South Central	5 (4.9)
West North Central	6 (5.9)
West South Central	11 (10.8)
Mountain	5 (4.9)
Pacific	13 (12.7)

Data presented as n (%).

## 4 Discussion

Despite the interest of residents applying for postgraduate fellowships, inadequate information is available on general trends within the application process, specifically for pain medicine fellowships. Applicants and training programs gauging the competitiveness of their application will still benefit from this information on the numbers of available positions and demographic trends [3]. Applicants are not awarded the same freedoms as medical students while they balance residency responsibilities with applying to secure a fellowship-training program after graduation. Applicants must carefully manage time, program cost, and additional stressors of travel while applying for fellowship [[12], [13], [14]]. This does not include the increasing responsibilities of life that come as one advances in age and career, such as starting a family or owning a home. The impact of the current fellowship match process in pain medicine is notable both in terms of expense and number of interviews undertaken by applicants and programs. For orthopedic fellowship interviews, the impact on the applicants and programs included missing 11 days of training and spending $5,875 on travel [13]. Similar concerns about the burden of a large quantity of interviews have been identified in general surgery where time away from training is usually covered by other residents as general surgery residents spent more than $4,000 on the interview process. Program directors rated fellowship burden as an average of 6.7 on a 1-to-10 scale of disruption, with 10 being a significant disruption [14]. The ease of attending the interview due to reduced travel costs can affect where applicants may attend interviews for fellowship training in pain medicine, and, ultimately, what fellowship they choose. Many fellowship programs may even have a preference to maintain their own internal applicants for ease of transition, or because they believe that they have a higher likelihood of coming to their program.

Additionally, while there continues to be an increase in pain medicine training positions, corresponding increases in overall applicants are lacking and may not be as sought-after as perceived. This may be in part due to poor pain medicine exposure at the medical student level. The need for a dedicated pain medicine curriculum for medical students is likely warranted and can be exemplified when examining the specialty of interventional radiology (IR) and its effect on increasing applicants [15]. The knowledge of IR clinical roles increased after the teaching session, which led to better understanding of IR and increased enthusiasm for pursuing the specialty as a future career. A similar initiative took place at Brown University with the advent of a pre-clinical pain medicine elective, which showed that first and second year students enrolled in the course had a better understanding of the field and were more likely to pursue a career in pain medicine after taking the course [16]. For a vast majority, exposure to specialties and indeed the timing of the exposure can significantly impact their future career choices [15].

Another salient finding is the discrepancy between male (77%) and female (23%) pain medicine fellows. A diverse, more representative healthcare workforce has been shown to improve performance and outcomes for patients [17]. In addition, population-based research consistently demonstrates greater pain prevalence among women relative to men [18]. Intuitively, this would suggest that a greater proportion of female pain physicians is necessary to better serve chronic pain patients. When considering the total proportion of male (80%) to female applicants (20%) - an even larger disparity between genders than matriculated fellows - this could suggest that programs are making an effort to counteract these gender disparities [3]. The male to female resident disparity in our research is more exaggerated than what is found in anesthesia programs, with females making up 37% of anesthesia residents [19]. To our knowledge, there are no published studies on Physical Medicine and Rehabilitation (PM&R) gender discrepancies, but the 2021 AAMC data on resident gender shows 35% of PM&R are female [20]. Although, our finding is similar to a prior study showing an increase of internal medicine female residents to 43.2% (2016) from 30.2% (1991). Interestingly, the same study found that between 1991 and 2016 female subspecialty fellows have decreased from 33.3% to 23.6% [21]. However, even if the data is the result of efforts made by program leadership and selection committees, clearly much more work must be done to offset this imbalance. This, in part, could be achieved with earlier exposure to the field for both men and women, as previously discussed, with a particular emphasis on this gender discrepancy.

The type of medical degree received by pain medicine fellows is also notable. A fellow’s medical degree may play a role within training programs, with 72.5% of fellows having a US medical degree, but only 55% of applicants having a US medical degree [3]. A higher proportion of US MD applicants matriculating into fellowship likely suggests that programs are preferential to applicants with US medical degrees. This could perhaps be due to the sheer quality of the applicant, confidence in the US MD-granting institution, or the preconceived notion that these fellows would be more likely to remain in the country or region than fellows from foreign medical institutions.

Our results should only be interpreted in the context of its limitations. The data in our study are strictly correlative, and so no definitive causal relationship can be made between any relationships, even if intuitive. For example, the fact that applicants are 20% female, but there is an increase to 23% matriculated fellows does not necessarily mean that programs are making concerted efforts to rank more female applicants to match. Another limitation stems from the likely variable nature of these statistics from year to year. As most of the data presented in this study are taken from one academic year, these relationships cannot be assumed constant nor predictive of future match characteristics.

To the best of our knowledge, this is the most relevant summary of the Pain Medicine Fellowship Match with respect to fellows, programs, and correlating characteristics. Although beyond the scope of this investigation, a survey-based design evaluating additional detailed information concerning websites, recruitment, program directors, selection committees, and applicants would be important to further explore these issues.[22] Further analysis of future NRMP ranking data may also provide valuable insights into understanding why these trends are occurring and how we can predict and change future patterns.

## 5 Conclusions

This study serves as a novel use of program data from multiple pain medicine departments to characterize several trends within the pain medicine fellowship match process. As the pain medicine application process remains competitive and the demand for pain physicians continues to grow, assessing which factors play a role in determining where and how applicants ultimately matriculate for fellowship is invaluable for individual applicants and programs. Overall, our analysis showed that geographical location plays an immense role in where applicants matriculate into fellowship, with a substantial percentage coming from the same state, region, and residency programs as their fellowship institutions. We also found that males and US MD applicants make up the bulk of both applicants and matriculated fellows. Finally, the number of fellowship positions offered in the past seven years has increased at a much higher rate than the corresponding applicant pool, and thus increased and earlier exposure to pain medicine is warranted to garner more interest in the field.

## Funding

The authors have no sources of funding to declare for this manuscript.

## Declaration of competing interest

The authors declare that they have no known competing financial interests or personal relationships that could have appeared to influence the work reported in this paper.
